# Probing the Dynamics of Solvation and Structure of the OH^-^ Ion in Aqueous Solution from Picosecond Transient Absorption Measurements

**DOI:** 10.3390/molecules15053366

**Published:** 2010-05-07

**Authors:** Olivier Poizat, Guy Buntinx

**Affiliations:** Laboratoire de Spectrochimie Infrarouge et Raman (UMR 8516 de l’Université et du CNRS), Centre d'Etudes et de Recherches Lasers et Applications (FR 2416 du CNRS), Bât. C5, Université des Sciences et Technologies de Lille, 59655 Villeneuve d'Ascq, France; E-Mail: guy.buntinx@univ-lille1.fr (G.B.)

**Keywords:** transient absorption spectroscopy, hydroxide ion solvation, solvation dynamics, bipyridine

## Abstract

The reaction of intracomplex proton transfer (44BPY**^-.^**...HO-H) → 44BPYH**^.^** + OH**^-^** that follows the photoreduction of 4,4’-bipyridine (44BPY) into its anion radical 44BPY**^-^** in the presence of 1,4-diazabicyclo[2.2.2]octane (DABCO) is investigated in acetonitrile-water mixtures by using picosecond transient absorption. The dependence of the appearance kinetics of the 44BPYH**^.^** radical on the water content reveals a highly diffusional proton transfer process that is controlled by the dynamics of solvation of the released hydroxide ion. The results are interpreted on the basis of a two-step mechanism where an intermediate solvation complex (44BPYH**^.^**)OH^-^(H_2_O)_3_ is formed first before evolving toward a final four-water hydration structure OH^-^(H_2_O)_4_.

## 1. Introduction

The nature and transport mechanism of the proton (H^+^) and hydroxide ion (OH^-^) in aqueous solution play a determinant role in a lot of areas related to acid-base chemistry, electrochemistry, catalysis, and biochemistry. These ions are strongly hydrated [1] and the hydrogen bonds in their solvation shell are continuously breaking and reforming due to thermal fluctuations. A concerted dynamical process of ion migration through the H-bond network arises via continuous interconversion between hydration complexes (Grotthuss chain mechanism [2,3]). This type of structural diffusion process is widely recognized as accounting for the anomalously high mobility of the H^+^ and OH^-^ ions [3,4]. Knowledge of the dominant ion hydration structures in aqueous solution is thus of crucial importance to the understanding of transport dynamics.

Numerous experimental [4,5,6,7] and theoretical [8,9,10,11,12,13,14,15,16,17,18,19,20,21] investigations have yielded a clear molecular-scale description of the H^+^ transport mechanism in acidic aqueous solution, based on fast interconversion between H_3_O^+^(H_2_O)_3_ and [H_2_O**…**H**…**H_2_O]^+^ complexes via proton transfer [10,17,18]. However, hydroxide ion mobility has received much less attention and the detailed solvation structure and transport mechanism is still the object of controversy. The solvated OH^-^ ion being a rather elusive particle that is not easily accessible to direct observation, very few experimental results are reported. In particular, femtosecond infrared experiments, which have been applied successfully to provide direct mechanistic insights into the migration of H^+^ [22,23,24], are almost useless in the case of OH^-^ since the broad infrared signatures of the hydrated OH^-^ are hidden by the strong absorption from H_2_O [25]. From a theoretical point of view, an earlier concept considers OH^-^ as a simple mirror image of H_3_O^+^, that is, a proton hole instead of an excess proton, with similar solvation shell topologies (3-fold coordination of the oxygen) and a transport mechanism directly inferred from the structural diffusion mechanism established for H_3_O^+^ by reversing hydrogen bond polarities [5,6,11,26,27]. This Lewis-type hydration configuration is supported by some experimental studies on microsolvated clusters in the gas phase [28,29,30] but has been claimed as improbable in bulk water on the basis of recent ab initio calculations [10,17,31]. Another theoretical analysis concludes that OH^-^ accepts three H bonds but donates one in addition, leading to a tetrahedral configuration again in agreement with the Lewis picture [32,33]. This solvation pattern has been suspected to lead to a considerably faster structural diffusion rate than that observed experimentally and, accordingly, considered as unphysical [34]. Finally, the theoretical picture that leads to the most realistic structural diffusion coefficient, recently introduced through a series of quantum molecular dynamics simulations [27,34,35,36,37], is that of a nonclassical, in the Lewis sense, hypercoordinated solvation complex in which OH^-^ is preferentially accepting four hydrogen bonds in a roughly planar OH^-^(H_2_O)_4_ arrangement, a weaker bond being possibly donated by the hydroxide hydrogen atom. This four-water hydration configuration of the hydroxide ion is strongly supported by neutron scattering [38,39], X-ray diffraction [40] and absorption [41], and photoelectron emission [42] experiments in bulk aqueous hydroxide solution. Ultrafast pump-probe spectroscopy has been used to characterize the time scale for hydroxide reorientation [43,44]. Most recently, the short-lived transient complex [HO…H…OH]^-^ predicted in the dynamical hypercoordination mechanism has been characterized from femtosecond pump-probe and 2D IR measurements of the O-H stretch of dilute HDO in D_2_O solutions of NaOD [45]. A comprehensive review of the existing literature on the solvation and diffusion of hydroxide in bulk water is provided in reference 37.

**Scheme 1:**
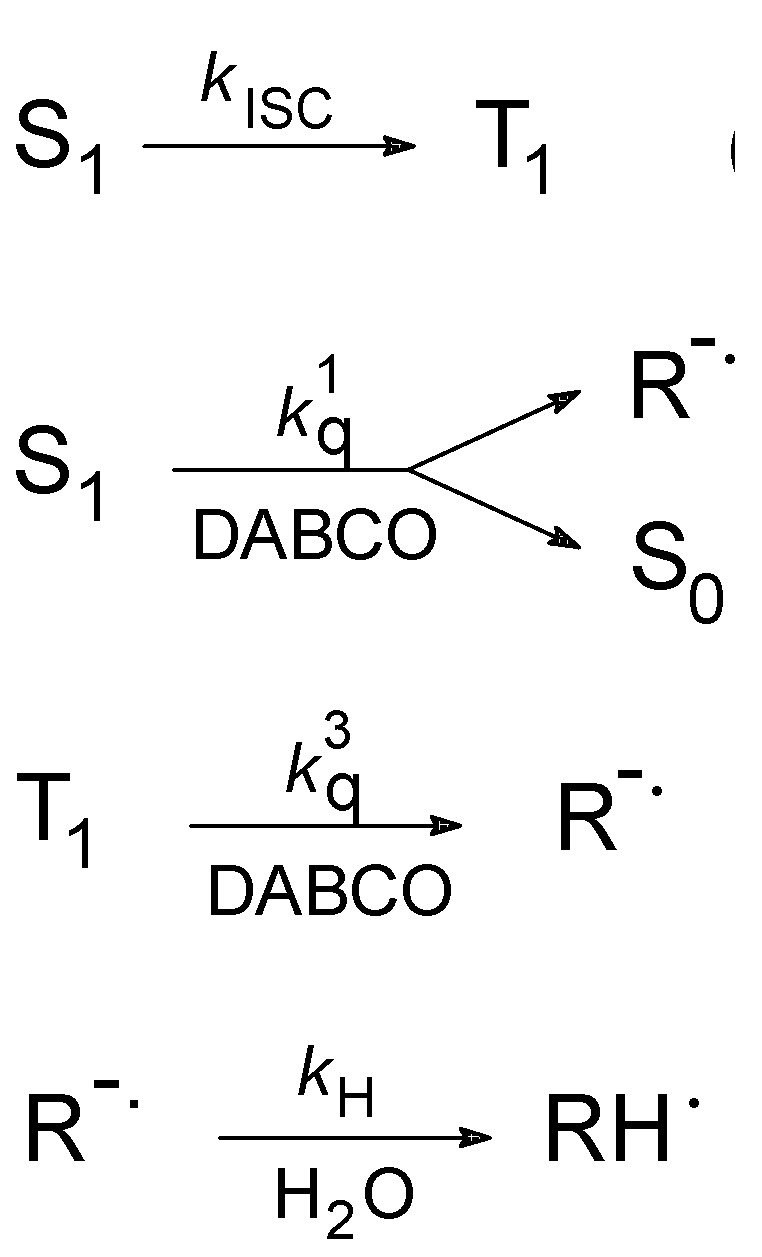
Photochemical reactivity of 44BPY in the presence of DABCO in aqueous solution (from Ref. 46). S_1_ = lowest exited singlet state, T_1_ = lowest exited triplet state, R^-^ = anion radical 44BPY**^-.^**, RH**^.^** = N-protonated radical 44BPYH.

The study presented in this paper is aimed at getting experimental information on the solvation dynamics of OH^-^ in aqueous solution by using time-resolved absorption spectroscopy. Since the OH^-^ ion has no significant absorption signature in the usual UV-visible region of investigation, we adopt an indirect approach that resides in probing a photochemical process that releases a hydroxide ion and for which the rate-limiting step in aqueous solution is the solvation of this ion. In a recent investigation of the photoreduction of the azaaromatic molecule 4,4’-bipyridine (44BPY) by amines, it was shown that the reduction product formed at both the excited singlet (S_1_) and triplet (T_1_) states, *i.e.* the anion radical 44BPY**^-.^**, is a strong photobase (pKa >15) that undergoes ultrafast N-protonation in aqueous solution [46] (see Scheme 1). 

The interesting point with regard to our purpose is that, in binary water – aprotic solvent mixtures [47], the parent 44BPY molecule as well as the photoproduced 44BPY**^-.^** ion were found to be preferentially solvated to water via hydrogen bonding, so that the 44BPY**^-.^** protonation by water must be a nondiffusional, intracomplex process: (44BPY**^-.^**...HO-H) → 44BPYH**^.^**+ OH**^-^**. Unexpectedly, the reaction rate measured as the rising kinetics of the 44BPYH**^.^** radical appeared to strongly increase with the water content in the solvent mixture, indicating some diffusional contribution to the process. Although these previous investigations [46,47] were mainly focussed on the photoreduction mechanism of 44BPY and on the nature and intrapair reactivity of the ion pair formed in this reaction, it was suggested that the diffusional character observed for the proton transfer process was due to a kinetic control of this process by the stabilization of the released hydroxide ion by solvation. Therefore, monitoring the appearance kinetics of the N-hydro radical product is expected to provide a direct measurement of the dynamics of OH**^-^** solvation. Having a spectral signature well-differentiated from that of the precursor anion radical [48], the 44BPYH**^.^** radical can be probed easily by transient absorption spectroscopy. This photochemical reaction is thus nicely suited for probing the solvation dynamics of the hydroxide ion in aqueous solution. The present report is essentially concerned with the dynamics of protonation of 44BPY**^-.^** by water in acetonirile-water solutions and its implication regarding the solvation dynamics of OH^-^ and the solvation structure of this ion.

## 2. Results and Discussion

**Figure 1:**
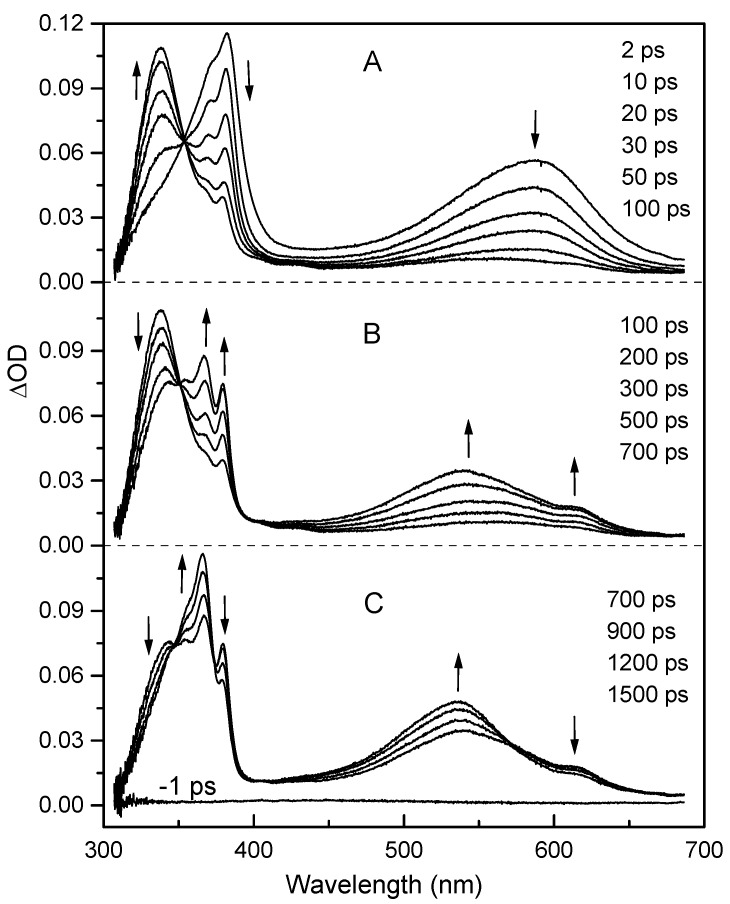
Transient absorption spectra of a solution of 44BPY (10^-2^ M) and DABCO (0.5 M) in a mixture of acetonitrile and 32% water by volume, at different time delay after 266 nm excitation: (A) 2-100 ps, (B) 100-700 ps, and (C) 700-1500 ps. Vertical arrows indicate the signal evolution.

We have probed by transient absorption spectroscopy the kinetics of protonation of the anion radical 44BPY**^-.^** produced upon photoreduction of 44BPY by the amine DABCO in a series of water-acetonitrile solutions. The water content in these solutions was varied from 10% to 100% by volume, which corresponds to the composition range in which the photoproduced anion is present in the form of an H-bonded complex (44BPY**^-.^**...HO-H) [47]. A typical example of time evolution of the absorption spectrum following excitation is shown in Figure 1 for a solvent mixture of acetonitrile and 32% water by volume. The observed spectral evolution is consistent with the results obtained previously in similar mixed solutions [47].

**Figure 2:**
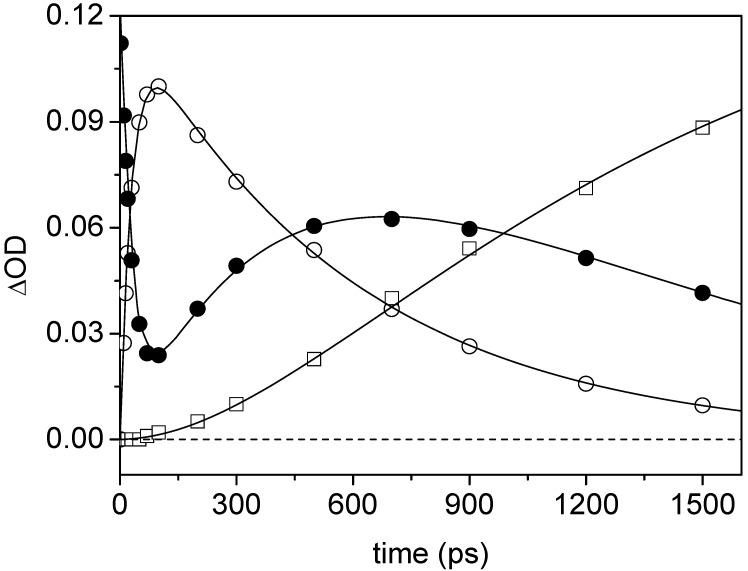
Time dependence of the extracted contributions of the T_1_ state absorption at 339 nm (ο), N-hydro radical absorption at 365 nm (□), and residual absorption (S_1_ state + anion radical) at 381 nm (●) and best fit (solid lines) obtained from the spectra in Figure 1.

The overall photoreduction scheme responsible for this spectral evolution has been well demonstrated [46,47]. It comprises three main steps that correspond roughly to the spectral evolution observed in the three time windows of Figure 1, respectively. In the 2-100 ps time range, the S_1_ state (λ_max_ = 381, 585 nm) leads to the T_1_ state (λ_max_ = 335 nm) by intersystem crossing (ISC). Then, in the 100-700 ps range, the T_1_ state is quenched by intermolecular electron transfer (ET) from the amine to yield the hydrated anion radical 44BPY**^-.^**...HO-H (λ_max_ = 380, 570, 610 nm) and the amine cation radical. Efficient reduction of the S_1_ state also takes place but is followed by an ultrafast back electron transfer that deactivates the reaction (Φ_deac_~0.95) [47]. Finally, after 700 ps, the extremely basic anion radical is rapidly protonated by water through the hydrogen bond and leads to the N-hydro radical 44BPYH**^.^** (λ_max_ = 360, 530 nm). However, one can see in Figure 1 that the ISC, ET, and PT reaction steps are not strictly separated in three distinct time domains and overlap more or less with each others. The kinetics of these processes are convoluted. The accurate determination of the proton transfer kinetics requires thus a global quantitative analysis of the kinetics of the overall reaction scheme. The treatment procedure has been described in detail previously [47]. Figure 2 shows the resulting kinetic analysis corresponding to the data in Figure 1 at three key wavelengths corresponding to the triplet state (339 nm), N-hydro radical (365 nm), and S_1_ state + anion radical (381 nm) absorption maxima. Excellent fits are found for all kinetics.

**Figure 3:**
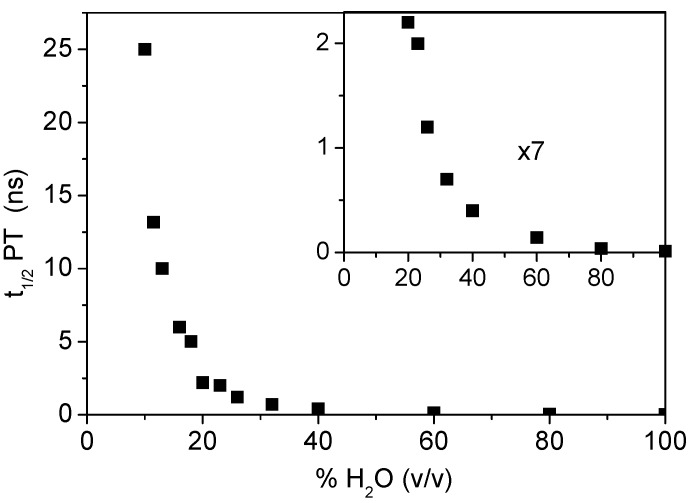
Variation of the characteristic time (ns) of protonation by water of the 44BPY^-^ anion radical as a function of the water content (% in volume) in acetonitrile-water solvent mixtures (DABCO concentration of 0.5 M).

**Figure 4:**
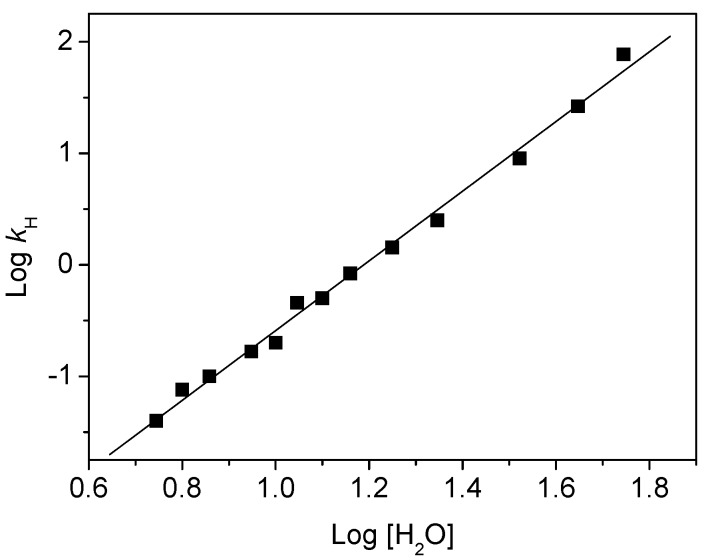
Variation of the rate constant *k*_H_ (in ns^-1^) of protonation by water of the 44BPY^-^ anion radical as a function of the water concentration (in M) in acetonitrile-water solvent mixtures (DABCO concentration of 0.5 M).

Similar kinetic analyses have been made for various solvent compositions and Figure 3 shows the dependence of the characteristic time τ_H_ determined for the proton transfer process (appearance time of the 44BPYH**^.^** radical) on the water content. One observes a continuous but highly nonlinear decrease of this time from 25 ns in a 10% water solution to 13 ps in pure water. Figure 4 shows the plot of the proton transfer rate constant (*k*_H_ = 1/τ_H_) *versus* the water concentration on a double-logarithmic scale. This plot is linear with a slope of 3.13 ± 0.2. It reveals a diffusion-controlled mechanism involving about three water molecules according to the law:

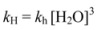
(1)
where *k*_h_ is a rate constant of quenching of 44BPY^-^ by proton abstraction. In other words, this diffusion law means that the presence of three water molecules in the vicinity of the (44BPY**^-^**...HO-H) entities is necessary to trigger the intracomplex proton transfer.

Finally, similar measurements have been done using D_2_O instead of H_2_O as cosolvent of acetonitrile in three solvent mixtures: 100% (pure water), 26%, and 13% water by volume, respectively. An approximately comparable slowdown of the intracomplex reaction is observed in all cases on going from proton transfer (rate constant k_H_) to deuteron transfer (k_D_), with a measured kinetic isotope effect of k_H_/k_D_ = 2.4 ± 0.3.

**Scheme 2:**
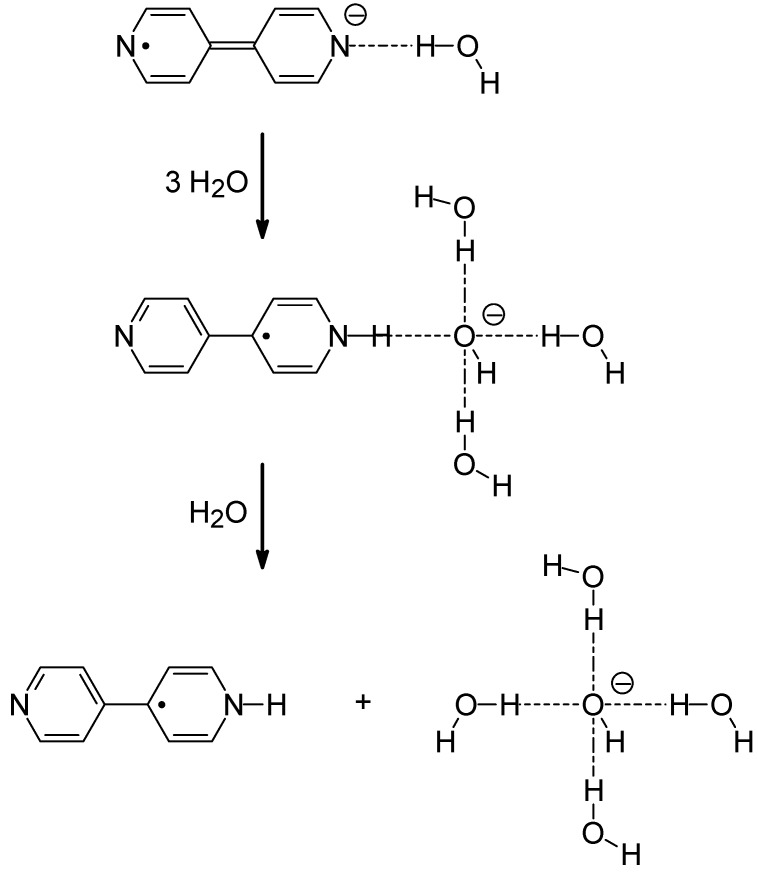
Two step reaction process proposed for the hydroxide ion solvation controlling the N-protonation of the 44BPY anion radical.

The above result showing that three water molecules are necessary for the intracomplex proton transfer process (44BPY**^-.^**...HO-H) → 44BPYH**^.^**+ OH**^-^** to take place implies that the rate-limiting step of the reaction is not the proton jump itself, which is inherently nondiffusional, but the stabilization of the released OH^-^ ion by solvation. The proton donor and acceptor are kept in contact by hydrogen bonding but the intracomplex proton jump is triggered by the diffusional approach of water molecules allowing to solvate the released OH^-^. The involvement of three water molecules in this solvation process could be simply interpreted, as previously suggested [47], as indicating that a three-water hydration configuration is the most stable solvation pattern of OH^-^ in aqueous solution, in support of the Lewis-type solvation picture [5,6,11,26,27]. However, this interpretation implicitly assumes that the equilibrium solvation structure of OH^-^ is reached as soon as the proton is transferred, without any intermediate step, which seems quite unlikely. In fact, immediately after intracomplex proton jump, the 44BPYH**^.^** and OH^-^ products are still in the close contact position of the H-bonded reactants before excitation and stay in contact to each other a certain time before separating, possibly also via H-bonding. During this short time, the bulky 44BPYH**^.^** radical belongs to the first solvation shell of OH^-^ and can be considered as playing the role of a solvation ligand in addition to the three water molecules required for the reaction. Therefore, despite the fact that only three water molecules are enough to trigger the proton jump by promoting the solvation of the released OH^-^, the first formed solvation structure of OH^-^ corresponds to a four-ligand configuration (44BPYH**^.^**)OH^-^(H_2_O)_3_. This result indicates that the real prerequisite for solvating the hydroxide ion is the formation of such a four-ligand configuration, which strongly supports the prediction that a four-water hydration structure OH^-^(H_2_O)_4_ is the dominant solvation pattern for OH^-^ in bulk water [31,34,35,36,37]. In the intracomplex proton transfer reaction studied in this work, the formation of this four-water hydration structure is probably a second step following the production of the initial and transient complex (44BPYH**^.^**)OH^-^(H_2_O)_3_ that is necessarily the first solvation structure of OH^-^ along the reaction pathway, according to the two-step process shown in Scheme 2 (the schematic representation of the 4,4’-bipyridine structure with quinoidal distortions symmetrically extended on the two pyridyl rings in the 44BPY**^-.^** anion radical and restricted to one pyridyl ring in the 44BPYH**^.^** radical conforms with previous time-resolved Raman and *ab initio* investigations of these transient species [49,50]). Both steps are controlled by the diffusion of water molecules but only the first step is rate-limiting for the proton transfer. By probing the kinetics of appearance of the 44BPYH**^.^** radical by proton transfer as a function of the water concentration, our measurements inform thus specifically on the dynamics of formation of the intermediate complex (44BPYH**^.^**)OH^-^(H_2_O)_3_, in agreement with the observed third order diffusional law *k*_H_ = *k*_h _[H_2_O]^3^, but does not provide any information on the second step dynamics, *i.e.,* the ligand exchange in the hydroxide solvation complex. The fact that, in acetonitrile-water solvent mixtures, the intracomplex H^+^ jump is driven by the diffusional approach of water molecules allowing to solvate the released OH^-^ can be understood in the framework of the presolvation concept introduced to describe the mechanism of OH^-^ migration in bulk water [34,37]. As soon as the donor water molecule is presolvated in a solvent cage appropriate to solvate OH^-^, the proton jump occurs. The proton jump and OH^-^ solvation events leading to the (44BPYH**^.^**)OH^-^(H_2_O)_3_ complex are much faster than the preceding process of diffusional migration of water molecules forming the presolvation pattern and thus cannot be resolved in our measurements. Indeed, H^+^ transfer and OH^-^ reorientation time constants reported from femtosecond pump-probe spectroscopy in aqueous hydroxide solutions at 295 K are faster than 3 ps [44,45].

## 3. Experimental

### 3.1. General

4,4’-Bipyridine (44BPY) and 1,4-diazabicyclo[2.2.2]octane (DABCO) were purchased from Aldrich. 44BPY was sublimed at 80 ºC *in vacuo* prior to each measurement. Acetonitrile (Prolabo, spectrophotometric grade) and DABCO were used as received. Water was distilled and deionized. Deuterium oxide (99.9 atom %D) was from Interchim. All measurements were performed on acetonitrile-water solutions of 44BPY (10^-2^ M) and DABCO (0.5 M). Photoexcitation of 44BPY was performed within the allowed S_0_→S_n_ (ππ*) transition of lowest energy (220–270-nm region) and followed by ultrafast internal conversion to a singlet S_1_ (nπ*) state [51,52].

### 3.2. Transient absorption measurements

The femtosecond transient absorption setup has been already described [51,52,53]. Briefly, it involves a 1 kHz Ti-sapphire laser system based upon a Coherent (MIRA 900D) oscillator and a BM Industries (ALPHA 1000) regenerative amplifier. Tripling the initial 90 fs pulses at 800 nm (0.3 mm BBO crystal) provided the pump excitation at 266 nm. Its power was limited to 10–20 µJ per pulse (1.0–2.0 mJ/cm^2^). A probe white light continuum pulse was generated at 800 nm in a CaF_2_ plate. The pump-probe polarization configuration was set at the magic angle. The probe pulse was delayed in time relative to the pump pulse using an optical delay line (Microcontrol Model MT160-250PP driven by an ITL09 controller, precision ±1 µm). The overall time resolution (fwhm of the pump-probe intensity cross-correlation) was estimated to be about 300 fs from the two-photon (pump + probe) absorption signal in pure hexane. The time dispersion of the continuum light over the 300–700 nm region of analysis was about 0.8 ps. The transmitted light was analyzed by a CCD optical multichannel analyzer (Princeton Instrument LN/CCD-1340/400-EB detector + ST-138 controller). Samples were circulating in a flow cell with 2.5 mm optical path length. Data were accumulated over 3 min (~180,000 pump-probe sequences).

## 4. Conclusions

Ultrafast generation of a super-photobase, the anion radical 44BPY**^-.^**, is achieved via electronic excitation of 44BPY and an electron donor in acetonitrile using femtosecond laser pulses. In the presence of water as co-solvent, 44BPY**^-.^** exists as an H-bonded complex and undergoes intracomplex proton abstraction (44BPY**^-.^**...HO-H) → 44BPYH**^.^**+ OH**^-^**. The proton transfer rate increases as the third power of the water concentration in the solution, which reveals the intervention of a diffusion-controlled process involving three water molecules. This result is interpreted as indicating that a presolvation cage appropriate to solvate the releasing OH^-^ must form around the proton donating water prior to the proton jump. Following this pre-reactional diffusion-controlled stage, fast intracomplex proton transfer process concomitant with hydroxide solvation in a four-ligand complex arises according to: (44BPY**^-.^**...HO-H) + 3 H_2_O → (44BPYH**^.^**)OH^-^(H_2_O)_3_. This first reaction step is assumed to evolve in a second step as: (44BPYH**^.^**)OH^-^(H_2_O)_3_ + H_2_O → 44BPYH**^.^** + OH^-^(H_2_O)_4_. This investigation by ultrafast pump-probe spectroscopy of the dynamics of OH^-^ solvation by water in acetonitrile-water solvent mixtures supports the picture of a four-water hydration configuration of the hydroxide ion in aqueous solution [27,34,35,36,37].
